# Pencil Graphite Electrodes Decorated with Platinum Nanoparticles as Efficient Electrocatalysts for Hydrogen Evolution Reaction

**DOI:** 10.3390/ma15010073

**Published:** 2021-12-23

**Authors:** Lorena-Cristina Balint, Iosif Hulka, Andrea Kellenberger

**Affiliations:** 1 Faculty of Industrial Chemistry and Environmental Engineering, Politehnica University Timisoara, Piata Victoriei 2, 300006 Timisoara, Romania; lorena.balint@gmail.com; 2 Research Institute for Renewable Energy, Politehnica University Timisoara, G. Musicescu nr. 138, 300501 Timisoara, Romania; iosif.hulka@upt.ro

**Keywords:** pencil graphite electrode, hydrogen evolution reaction, platinum nanoparticles, pulsed current electrodeposition, water electrolysis

## Abstract

Platinum-based materials are widely known as the most utilized and advanced catalysts for hydrogen evolution reaction. For this reason, several studies have reported alternative methods of incorporating this metal into more economical electrodes with a carbon-based support material. Herein, we report on the performance of pencil graphite electrodes decorated with electrochemically deposited platinum nanoparticles as efficient electrocatalysts for hydrogen evolution reaction. The electrodeposition of platinum was performed via pulsed current electrodeposition and the effect of current density on the electrocatalytic activity was investigated. The obtained electrodes were characterized using cyclic voltammetry, while the electrocatalytic activity was assessed through linear sweep voltammetry. Field emission scanning electron microscopy and energy-dispersive X-ray spectroscopy were utilised to gain an insight into surface morphology and chemical analysis of platinum nanoparticles. The best performing electrocatalyst, at both low and high current densities, was characterized by the highest exchange current density of 1.98 mA cm^−2^ and an ultralow overpotential of 43 mV at a current density of 10 mA cm^−2^. The results show that, at low current densities, performances closest to that of platinum can be achieved even with an ultralow loading of 50 µg cm^−2^ Pt.

## 1. Introduction

Platinum-based materials are still state of the art electrocatalysts for the hydrogen evolution reaction (HER), intensifying the cathodic reaction and possessing a good stability in the corrosive acid environment. Because the electrochemical reactions take place on the surface of the catalyst particles, an active surface as large as possible at the catalytic layer of the electrode is required [1]. For this reason, the deposition of catalyst nanoparticles on the surface of carbon-based support materials is a reference model, due to their very high specific surface area. The carbon-based materials that can be used as a support for platinum deposition are carbon nanofibers, carbon nanotubes, carbon nanoparticles (carbon black—CB) and/or graphene oxide [2,3,4,5,6,7,8]. Furthermore, many data have been recorded for carbon nanostructures as a platinum-supporting material, emphasizing their contribution to electrocatalyst performances. All studies summarize an improved electrocatalytic activity, with a higher electrochemical surface area [5,7]. The interaction between carbon-based supports and platinum catalysts presents such a large interest due to their ability to balance each other’s deficiencies. Firstly, a carbon support with suitable properties is required in order to assure an active electrocatalyst and to compensate for the economically feasibility platinum lacks [2]. However, it is clear that carbon alone cannot fulfill the catalytic requirements, and therefore platinum deposition plays a vital role in assuring the electrocatalytic activity. The main challenge is to achieve a catalyst loading as low as possible, while still preserving the electrocatalytic properties. Currently, the catalytic loading is maintained in a range of 0.5–1 mg Pt/cm^2^, further decreases being still necessary down to potential values reaching below 0.2 mg cm^−2^ [9]. Decreasing the catalytic loading can be achieved by using appropriate deposition methods, which enables the formation of nanoparticles with a narow size distribution, highly dispersed on the support surface, with a high utilization degree of the catalyst particles. A promising technique in this sense is the pulsed current electrodeposition [10,11], which implies an alternated shift of the current between two different values, usually the applied current during the on-time and a zero current during the off-time. These pulses favour a faster nucleation rate and greatly increase the number of grain nuclei per unit area, resulting in finer grained deposits with better properties than those obtained by direct current electrodeposition [12]. When changing from a direct to a pulsed current, the average size of Pt nanoparticles is decreased and their surface becomes more rough, which is beneficial for the electrocatalytic activity [13].

Over recent years, graphite in the form of pencil lead has gained a large applicability as a voltammetric electrode, especially in electroanalysis [14,15,16], due to several advantages. Pencil graphite (PG) electrodes overcome the problems raised with the use of other carbon-based supports, which usually require mechanical surface finishing; this is not only time consuming, but is also threatening to the crystal structure of the graphite. Since PG is very affordable and availability is not a concern, it can be used as a disposable electrode. Since the quality of the pencil graphite is determined in the highly controlled manufacturing process, a uniform composition and surface is guaranteed, ensuring the reproducibility of the results. Furthermore, these electrodes are described by sensitivity, and are considered a viable, renewable and economical alternative [14]. Their high activity, stability and cost-efficiency has boosted the interest of their use for other important electrochemical processes, such as hydrogen evolution reaction [17].

In this work, we report on activated pencil graphite electrodes decorated with Pt nanoparticles, as an efficient electrocatalyst for hydrogen evolution reaction in acid environments. We investigate the effect of current density during pulsed current electrodeposition of Pt on the electrocatalyst performance for HER. Cyclic voltammetry (CV) in 0.5 M H_2_SO_4_ is first used as validation for the presence of Pt nanoparticles on the surface of the carbon-based support, as well as for the electrochemical characterization, while linear sweep voltammetry (LSV) was used to gain an impression of the electrocatalytic activity of Pt-PG electrodes for HER. Surface morphology and chemical analysis of Pt nanoparticles were performed using field emission scanning electron microscopy (FE-SEM) and energy-dispersive X-ray spectroscopy. A quantitive assessment for the electrocatalytic activity for HER was made using the kinetic parameters extracted from the Tafel plots. Another important parameter used to compare electrocatalytic activity was the overpotential required to reach 10, 50 and 100 mA cm^−2^ for all Pt-PG electrocatalysts. Furthermore, stability tests of the electrodes consisting of carbon-supported Pt nanoparticles were performed by chronopotentiometry.

## 2. Materials and Methods

### 2.1. Preparation of Pt-PGE Electrodes

Platinum electrodeposition on pencil graphite electrodes was carried out by a current pulse method, from a solution of 5 mM H_2_PtCl_6_ (Sigma Aldrich, Darmstadt, Germany >99.9%) in 1 M HCl (Merck, ACS, ISO, Reag PhEur, p.a. 37%). For the deposition, a typical three-electrode system was used, with a silver—silver chloride reference electrode (Ag/AgCl, KCl 3M, BASi^®^, West Lafayette, IN, USA, a Pt wire counter electrode and a working electrode consisting of pencil graphite (pencil lead 0.7 mm B, 99% C) with an exposed length of 20 mm. The current pulse was applied using an Autolab PGSTAT 302N potentiostat/galvanostat (Metrohm Autolab, Utrecht, The Netherlands. Several electrodes were prepared, by varying the current density (50, 25, 10 and 5 mA cm^−2^) and maintaining constant the other deposition parameters, on- and off-time and duty cycle (*t*_on_ = 0.1 s, *t*_off_ = 0.5 s, duty cycle 16.67%, number of cycles = 200).

Prior to use the PG electrodes, an electrochemical pre-treatment step is required to activate and stabilize the electrode surface. Usually, PG activation is accomplished by electrochemical oxidation. This electrochemical treatment modifies the surface properties by generating surface defects, which improve the kinetics of electron transfer [15,16]. PG activation was performed by cyclic voltammetry (CV), applying 10 scans from −0.30 V to 2.00 V vs. Ag/AgCl, at a scan rate of 100 mV s^−1^, in 0.5 M H_2_SO_4_ solution (Merck, 95–97%, p.a.). The activated PG electrodes are denoted by PG*. The activation of PG electrodes, and generally of carbon-based materials, has the effect of generating reactive functional groups on the surface, usually carbonyl and/or carboxyl groups, which act as reactive centers in their interaction with various molecules. Another effect of activation is the marked increase in capacitance and specific surface area, which are also important parameters for materials used as electrodes for the hydrogen evolution reaction.

### 2.2. Electrochemical and Physico-Chemical Characterization of Activated PG and Pt-PG Electrodes

All electrochemical measurements were performed using an Autolab 302N potentiostat/galvanostat (Metrohm Autolab, Utrecht, The Netherlands) and the previously described electrochemical cell configuration. Electrode potentials measured versus the Ag/AgCl reference electrodes were converted to potentials versus reversible hydrogen electrode (RHE) using the relation: *E* vs. RHE = *E* vs. Ag/AgCl + 0.059pH + *E*_Ag/AgCl_. Cyclic voltammetry was used to determine the double layer capacitance of activated PG* electrodes, in 0.5 M H_2_SO_4_ solution at different scan rates, from 10 to 200 mV s^−1^. After Pt electrodeposition, the freshly prepared Pt-PG electrodes were rinsed with distilled water, transferred to a 0.5 M H_2_SO_4_ solution and cycled between 0.00 and 1.45 V vs. RHE for 200 scans, until a stable electrochemical response was recorded. The last scan was used to calculate the electrochemical surface area (ECSA) of each electrocatalyst, by computing the charge necessary for the H adsorption process.

The structure and morphology of PG and Pt-PG electrodes have been characterized by field emission scanning electron microscopy (FE-SEM) using a QUANTA FEG 250 microscope (Quanta FEG 250, FEI, Hillsboro, OR, USA), using a secondary electron detector (SE), and a pressure-limiting aperture (PLA). The studies were performed in a low vacuum mode in order to minimize the charging of the samples using an accelerating voltage of 15 or 20 kV and a working distance of 10 mm. The elemental composition was determined by energy dispersive X-ray analysis (EDX with Apollo SSD detector, EDAX Inc., Mahwah, NJ, USA) using a pressure limiting aperture (PLA). PLA is used for analysis by positioning the samples at 10 mm working distance, where the stage is at eucentric height. The longer profile of the PLA minimizes the low voltage beam dispersion and skirting of the primary beam in the gaseous environment of the chamber, allowing more electrons to interact with the specimen when focused and increasing the signal-to-noise ratio. This setup allows the collection of EDX spectra of the highly charged particles.

### 2.3. Electrochemical Measurements for Hydrogen Evolution Reaction

Electrocatalytic activity for HER was evaluated by linear sweep voltammetry in 0.5 M H_2_SO_4_ and compared to a commercial Pt disk electrode (99.95% Pt, BASi^®^, 3.0 mm). The reproducibility was checked by recording three consecutive linear voltammograms. Kinetic parameters (*b*—Tafel slope and *i_o_*—exchange current density) were derived from the Tafel plots. Stability tests were carried out by chronopotentiometry in 0.5 M H_2_SO_4_ at a current density of 10 mA cm^−2^ for 6 h.

## 3. Results and Discussion

**Figure 1:**
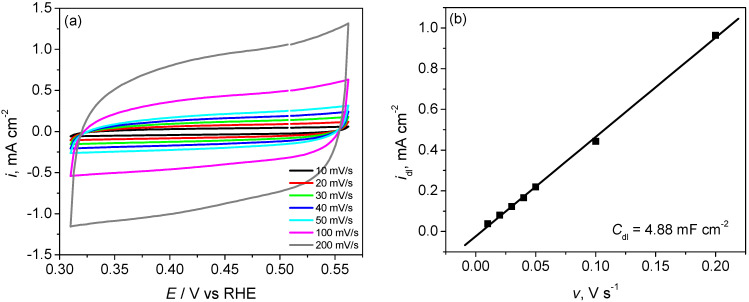
(**a**) Cyclic voltammograms for activated PG* electrodes in 0.5 M H_2_SO_4_ solution at different scan rates; (**b**) Dependence of the capacitive current density on the scan rate.

Cyclic voltammograms of activated PG* electrodes given in Figure 1a were used to evaluate the double layer capacitance. CVs recorded at different scan rates show a capacitive behaviour, characterized by a square-shaped CV, without peaks corresponding to an electrochemical reaction, and so the observed current corresponds only to double layer charging. The capacitance of the double layer *C*_dl_ was calculated using Equation (1) [18]:

(1) $$C_{dl}=\frac{\partial Q}{\partial E}=\frac{I\cdot dt}{dE}=\frac{I}{v}\;$$

The plot of the capacitive current density *i*_dl_ obtained at an electrode potential of 0.45 V versus the scan rate, and gives a straight line as indicated in Figure 1b, with a slope equal to the double layer capacitance *C*_dl_. The value of the double layer capacitance for the activated PG* electrode is 4.88 mF cm^−2^. Compared to the frequently used value of 20 µF cm^−2^ for a perfectly smooth metal surface in contact with an electrolyte solution of ordinary concentration [18], an increase of about 200 times of the specific surface can be estimated. These results indicate an important increase of double layer capacitance and specific surface of activated PG* electrodes, parameters that are important for developing highly efficient electrocatalysts for HER.

**Figure 2:**
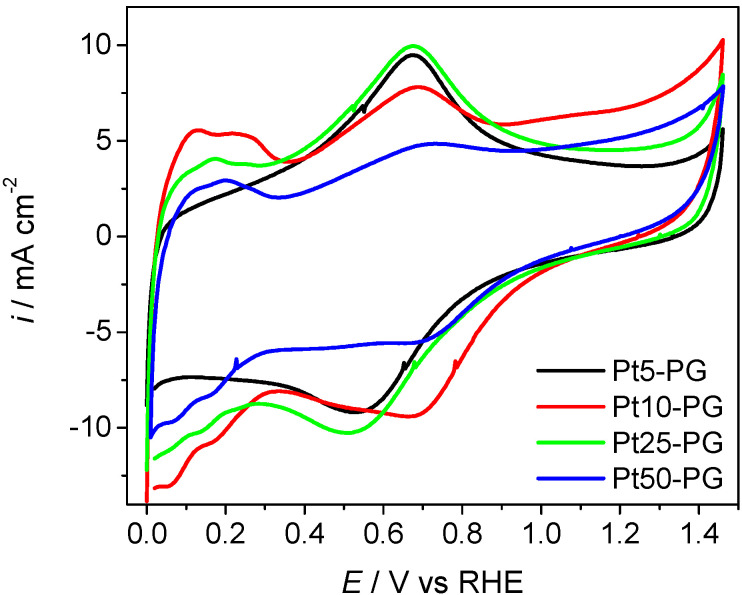
Cyclic voltammograms for ECSA determination (200th scan) of Pt-PG electrodes in 0.5 M H_2_SO_4_ solution, scan rate 500 mV s^−1^.

The activated PG* electrodes were used for Pt electrodeposition by a pulsed current method and the electrodeposition parameters are summarized in Table 1. Platinum electrodeposition on activated PG* was confirmed by recording the cyclic voltammograms in 0.5 M H_2_SO_4_, as shown in Figure 2. The CVs are characterized by the presence of two oxidation/reduction peaks at electrode potentials of 0.11 and 0.22 V, corresponding to the adsorbed hydrogen, respectively and an oxidation/reduction peak at 0.68 V due to the formation and reduction of Pt oxide at the surface of the electrode. The appearance of the characteristic peaks of hydrogen adsorption/desorption processes, respectively, of platinum oxide formation, is an indication that platinum deposition took place. The CV of Pt5-PG, the electrode obtained by Pt deposition at the lowest current density, shows only the presence of the oxidation/reduction peak of Pt oxide, whilst the peaks characteristic of hydrogen adsorption/desorption are absent, which can be explained by the deposition of very small amounts of Pt.

The cyclic voltamograms in Figure 2 were used to calculate the active electrochemical surface area (ECSA) according to Equation (2). For the calculation of ECSA, the voltamogram part corresponding to the hydrogen adsorption/desorption region between 0 and 0.3 V was used. This region is integrated and the contribution of the capacity of the double layer was subtracted, obtaining the amount of electricity used for hydrogen desorption [19,20].

(2) $$ECSA=\frac{Q_{H,des}}{m_{Pt}\times Q_{mono}}=\frac{\frac{1}{v}\int^{\;}i\left(E\right)dE}{m_{Pt}\times Q_{mono}}$$

where: *Q*_H,des_ represents the charge needed for hydrogen desorption (μC), *m*_Pt_ is the amount of platinum deposited, *Q*_mono_ is the charge required for the adsorption of a monoatomic layer of hydrogen on the surface of the polycrystalline platinum electrode (210 μC cm^−2^) [21], *i*(*E*) is the current (μA), *v* is the scan rate (V s^−1^) and *E* represents the electrode potential (V).

The amount of electrodeposited Pt was estimated using Faraday’s law of electrolysis, assuming a 100% current efficiency, according to Equation (3):

(3) $$m_{Pt}=\frac{A_{Pt}}{z\cdot F}\cdot I\cdot t_{on}\cdot n$$

where: *A*_Pt_ is the atomic mass of platinum (g mol^−1^); *z* is the number of electrons used for the reduction; *F* is the Faraday’s constant (96,485 C mol^−1^); *I* the current intensity applied during pulse electrodeposition (A); *t*_on_ is the pulse duration (s) and *n* represents the number of cycles.

Using the determined ESCA values, an estimation of the average size of the Pt nanoparticles can be realized according to Equation (4).

(4) $$d=\frac{6}{\rho \cdot ESCA}$$

where ρ is the platinum density (g m^−3^) and *d* is the mean diameter of the Pt particles. This equation assumes individual particles, with a spherical geometry and the mean diameter calculated from the ratio between the volume and surface of a sphere. Table 1 summarizes the obtained catalyst loading, electrochemical surface area and particle size of Pt deposited on PG*, by a pulsed current deposition, at different current densities. As expected, the platinum loading increases with the current density used during the pulse electrodeposition, and the obtained values are within values reported in the literature. The electrochemical surface area also depends on the current density during electrodeposition and the highest value is obtained for a current density of 10 mA cm^−2^. The particle size calculated from ECSA shows that the expected particle size is in the order of hundreds of nanometers and increases with the increasing current density applied during pulse electrodeposition.

**Figure 3:**
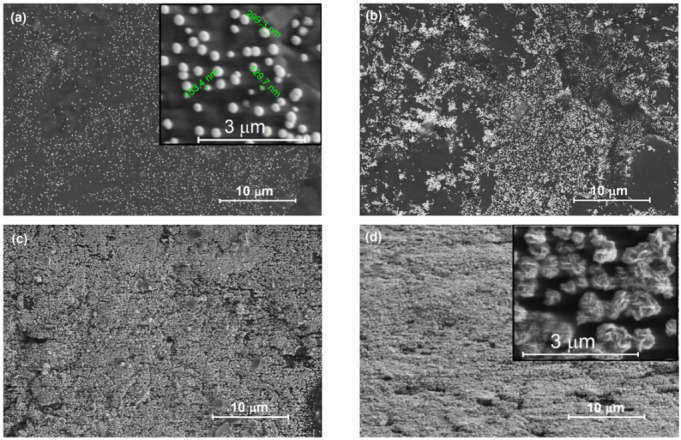
SEM micrographs of Pt-PG electrodes obtained by pulsed current deposition from 5 mM H_2_PtCl_6_ in 1 M HCl solution, at different current densities: 5 mA cm^−2^ (**a**); 10 mA cm^−2^ (**b**); 25 mA cm^−2^ (**c**); and 50 mA cm^−2^ (**d**). Insets show a higher magnification of Pt5-PG and Pt50-PG structures. The overall deposition time in all cases was 120 s.

Figure 3 shows SEM images of the surface morphology of Pt deposited on PG* by pulsed current deposition with current densities of 5, 10, 25 and respectively 50 mA cm^−2^.

The SEM micrographs reveal that Pt nanoparticles are homogeneously distributed on the PG surface. Applying a low current density of 5 mA cm^−2^ during electrodeposition results in well dispersed, single catalyst nanoparticles, with spherical shape and dimensions of around 130–300 nm. The Pt nanoparticles increase up to a limited dimension, and afterwards the nucleation/growth takes place on adjacent sites. A similar self-limiting growth mechanism has also been reported for Pt nanoparticles electrodeposition at a constant current [8]. Increasing the applied current density to 10 mA cm^−2^ promotes the formation of catalyst nanoparticle clusters, while still maintaining the sherical morphology. A further increase in current density to 25 and 50 mA cm^−2^ resulted in a marked increase of the electrodeposited platinum quantity, which forms a porous layer completely covering the carbon-based substrate, but is more important to a change of morphology. Figure 3d and the higher magnification FE-SEM image, shown as inset, indicate that the platinum layer is predominantly characterized by a dendritic flower-like morphology, with structures’ dimensions of several hundreds of nm. These findings are very similar to the change in morphology that has been observed for Pt nanoparticles deposited on carbon paper under galvanostatic conditions using a direct current [8,22] instead of a pulsed current. A gradual transition of Pt nanoparticle morphology from a spherical to a flower-like and dendritic structure has been also reported during pulsed potential electrodeposition of Pt [23]. This is an evidence that nucleation/growth and morphology of Pt nanoparticles can be controlled by the current density during electrodeposition. Moreover, when compared to direct current electrodeposition, pulsed current electrodeposition increases the nucleation rate, resulting in smaller sized particles [13]. Another remarkable advantage of pulse electrodeposition is the shorter deposition time.

**Figure 4:**
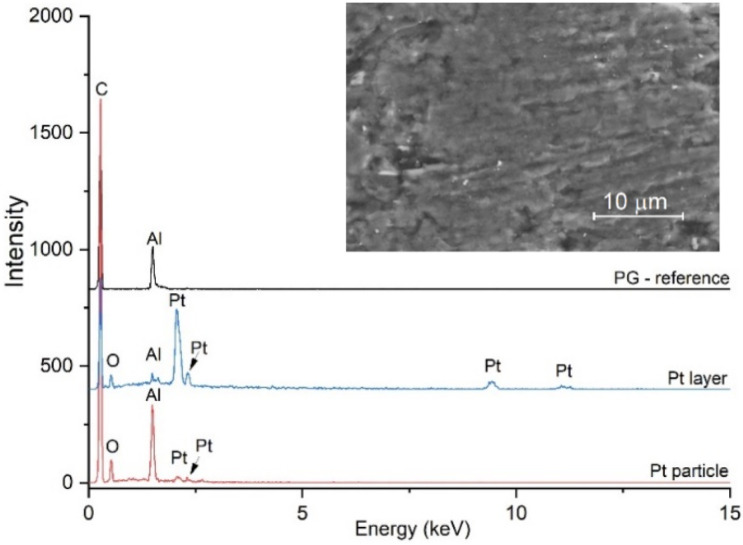
EDX analysis; spectra collected on the untreated PG electrode, on the Pt layer (Pt50-PG) and on a single Pt particle (Pt5-PG). Inset shows an SEM micrograph of the untreated PG electrode.

The chemical composition determined by EDX is shown in Figure 4 and indicates the presence of C (99.51 wt%) and Al (0.49 wt%) as an impurity in the untreated PG, which is constantly found for all samples. For Pt-PG electrodes, the composition of Pt depends on the current density. EDX analysis on a single Pt particle (Pt5-PG) indicate an average Pt content of about 1 wt%, while on the Pt layer the detected average content increases to 9.6 wt% (Pt25-PG) and 12 wt% (Pt50-PG) respectively. Moreover, the presence of oxygen is detected, which is attributed to the organic functional groups generated on the PG surface during the electrochemical oxidation/activation step.

**Figure 5:**
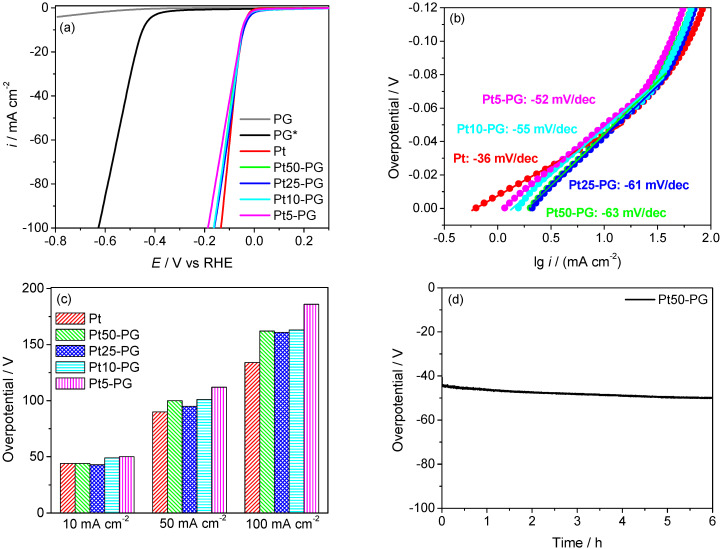
(**a**) Polarization curves of un-treated PG, activated PG*, Pt and Pt-PG electrodes in 0.5 M H_2_SO_4_ solution at a scan rate of 50 mV s^−1^. (**b**) Tafel plots of Pt and Pt-PG electrodes. (**c**) Required overpotential to reach a current density of 10, 50 and 100 mA cm^−2^ for Pt and Pt-PG electrodes. (**d**) Chronopotentiometric stability test of Pt50-PG at a current density of 10 mA cm^−2^.

The electrocatalytic activity for HER was investigated for the untreated and activated PG*, as well as for the Pt-PG electrodes obtained by electrodeposition of Pt at different current densities and compared to a commercial Pt electrode. The linear polarization curves and the correponding Tafel plots are given in Figure 5*,* together with the overpotential values necessary to reach a current density of 10, 50 and 100 mA cm^−2^ and the stability test at constant current density of 10 mA cm^−2^.

Linear polarization curves in Figure 5a show that the un-treated PG has no electrocatalytic activity for hydrogen evolution, as indicated by the large overpotential of 513 mV at a current density of 1 mA cm^−2^. The activation of PG by electrochemical oxidation induces a significant increase in electrocatalytic activity for HER, but the overpotential is still about 400 mV higher than on the commercial Pt electrode. In contrast, all Pt-PG electrodes exhibit an electrocatalytic activity identical to that of the Pt electrode at lower current densities (<40 mA cm^−2^). At higher current densities (100 mA cm^−2^), a slight increase of the overpotential is observed, by 20 mV for Pt-PG electrodes obtained at 50, 25 and 10 mA cm^−2^ and by 50 mV for Pt-PG electrodeposited at 5 mA cm^−2^. For the quantitative assessment of the electrocatalytic activity for HER, the kinetic parameters obtained from the Tafel plots shown in Figure 5b were used. The values of the exchange current density *i*_o_ and Tafel slope *b* are summarized in Table 2, together with the overpotential values at 10 and respectively 100 mA cm^−2^.

It is generally accepted that HER kinetics on polycrystalline Pt in acid media is overpotential-dependent [24,25,26]. At low overpotentials, the initial discharge step (Volmer reaction) is fast and the recombination (Tafel reaction) is a rate-determining step (rds) leading to a Tafel slope *b* ~30 mV/decade. At higher overpotentials, the coverage of adsorbed hydrogen atoms approaches saturation, so the recombination step becomes faster and the discharge step becomes rds, with a measured Tafel slope *b* ~120 mV/decade [27]. Our experimental results are in line with this mechanism, showing the presence of two distinct Tafel slopes. The value of the exchange current density *i_o_* describes the reaction rate at the equilibrium potential. For all Pt-PG electrodes, the values of exchange current density are higher when compared to Pt. However, these are apparent values, correlated with the increased specific surface area of the electrodes. It is interesting to note that at a low overpotential, the Tafel slopes of flower-like Pt particles (Pt25-PG and Pt50-PG) are higher that those of Pt particles with a spherical morphology (Pt5-PG and Pt10-PG) but the trend is reversed at higher overpotentials. This behaviour, corellated with higher exchange current densities, make flower-like morphology catalyst particles outperform hemispherical ones.

The overpotential necessary to attain a certain current density is another important parameter, practically used to evaluate the electrochemical performance of electrocatalysts during operation. Usually, for HER, overpotentials needed to reach 10 or 100 mA cm^−2^ are frequently reported. Figure 5 gives on overview of the ovepotentials needed to reach 10, 50 and 100 mA cm^−2^ for all Pt-PG electrocatalysts. It is remarkable that Pt25-PG requires an ultralow overpotential of 43 mV to achieve a current density of 10 mA cm^−2^, lower than that of Pt10-PG (49 mV) and of Pt5-PG (50 mV) and comparable to the overpotential of bulk Pt (44 mV). Moreover, at larger cathodic current densities of 50 and 100 mA cm^−2^, the Pt25-PG electrocatalyst shows superior performance (95 and 161 mV, Figure 5c) compared to Pt10-PG (101 and 163 mV) and Pt5-PG (112 and 186 mV). The enhanced electrocatalytic activity can be attributed to an optimum catalyst loading and a favourable morphology of the catalyst particles. Even so, all electrocatalysts showed good performances for HER, especially at low current densities. Pt5-PG, the electrode with the lowest catalytic loading, shows only a 16% increase of the overpotential as compared to Pt25-PG. Stability tests conducted at constant current density of 10 mA cm^−2^ are shown in Figure 5d and reveal that the overpotential remains almost stable, with a low shift of 5.4 mV in 6 h. Table 3 shows a comparision of the electrocatalytic performances for HER of Pt nanoparticles deposited on various carbon substrates by electrochemical and chemical deposition methods.

Pt-PG electrocatalysts developed in this work show performances within the range reported in the literature. In terms of the overpotential, Pt25-PG is superior to similar electrocatalysts obtained by current pulse or constant potential electrodeposition. The advantages of Pt-PG electrocatalysts rely on the availability and cost-efficiency of the substrate and the simplicity and rapidity of the current pulse electrodeposition method.

## 4. Conclusions

We have successfully used pencil graphite electrodes to synthesize low Pt loading electrocatalysts for hydrogen evolution reaction by a pulsed current electrodeposition method. It has been shown that current density during pulse electrodeposition plays an important role in controlling the morphology of Pt particles. Deposition at a low current density leads to spherical particles, and then the morphology gradually changes to flower-like structures as the current density increases. The electrocatalytic activity towards hydrogen evolution reaction is affected by both morphology and catalyst loading. The electrode with 250 µg cm^−2^ Pt loading revealed the best electrocatalytic activity, at both low and high current densities, needing only an ultralow overpotential of 43 mV to reach a current density of 10 mA cm^−2^. Still, decreasing the catalyst loading to 50 µg cm^−2^ Pt, a loss of 16% of the electrocatalytic activity is obtained. The results emphasize the existence of a trade-off between electrocatalytic activity and catalyst loading and cost.

## Figures and Tables

**Table 1:** Catalyst loading, electrochemical surface area ECSA and particle size of Pt-PG electrodes obtained by pulsed current electrodeposition in 5 mM H_2_PtCl_6_ in 1 M HCl solution, at different current densities.

Electrode	Pulse Electrodeposition Parameters (Current Density, *t*_on_, *t*_off_, Number of Cycles)	Pt Loading(mg cm^−2^)	ECSA(m^2^ g^−1^)	Particle Size * (nm)
Pt50-PG	50 mA cm^−2^; 0.1 s; 0.5 s; 200	0.50	1.36	206
Pt25-PG	25 mA cm^−2^; 0.1 s; 0.5 s; 200	0.25	2.15	130
Pt10-PG	10 mA cm^−2^; 0.1 s; 0.5 s; 200	0.10	3.64	77
Pt5-PG	5 mA cm^−2^; 0.1 s; 0.5 s; 200	0.05	-	-

* Estimated from ECSA values.

**Table 2:** Kinetic parameters for HER on Pt, Pt-PG and activated PG* electrodes in 0.5 M H_2_SO_4_.

Electrode	*i*_o_ (mA cm^−^^2^)	*b* (mV) low *η*	*b* (mV) high *η*	*η* @ 10 mA cm^−2^	*η* @ 100 mA cm^−2^
Pt	0.62	−36	−120	−44	−134
Pt50−PG	1.95	−63	−125	−44	−162
Pt25−PG	1.98	−61	−130	−43	−161
Pt10−PG	1.38	−55	−133	−49	−163
Pt5−PG	1.12	−52	−163	−50	−186
PG*	1.7 × 10^−4^	−94	−120	−450	−627

**Table 3:** Comparative data on the electrocatalytic performance for HER of Pt nanoparticles.

Catalyst	Preparation Method	*η* @ 10 mA cm^−2^ (mV)	*b* (mV decade^−1^)	Ref.
Pt25-PG	Current pulse electrodeposition on PG	43	61	This work
PtCoMn-CNT	Current pulse electrodeposition	N/A	123	[20]
10Pt@HN-BC	Constant potential electrodeposition	47	35	[28]
Pt-CNT	Ultra-low temperature reduction	41	49	[29]
Pt@GO@Ni-Cu@NF	Constant potential electrodeposition	31	51	[30]
PtNPs/CNFs	Electrospinning/carbonization	175	50	[31]
Pt/NCNTs	Chemical reduction	40	33	[32]
Pt-CQDs/Gr-C400	Electrolysis-solvothermal	38	40	[33]

## Data Availability

Not applicable.
